# Diagnostic Accuracy of Detecting Diabetic Retinopathy by Using Digital Fundus Photographs in the Peripheral Health Facilities of Bangladesh: Validation Study

**DOI:** 10.2196/23538

**Published:** 2021-03-09

**Authors:** Tahmina Begum, Aminur Rahman, Dilruba Nomani, Abdullah Mamun, Alayne Adams, Shafiqul Islam, Zara Khair, Zareen Khair, Iqbal Anwar

**Affiliations:** 1 Institute for Social Science Research The University of Queensland Brisbane Australia; 2 icddr,b Dhaka Bangladesh; 3 McGill University Montreal, QC Canada; 4 Barisal Medical College Hospial Barisal Bangladesh; 5 The Fred Hollow Foundation Dhaka Bangladesh

**Keywords:** diabetic retinopathy, diagnostic accuracy, digital fundus photograph, Bangladesh, diabetes, retinopathy, retina, opthalmology

## Abstract

**Background:**

Diabetic retinopathy can cause blindness even in the absence of symptoms. Although routine eye screening remains the mainstay of diabetic retinopathy treatment and it can prevent 95% of blindness, this screening is not available in many low- and middle-income countries even though these countries contribute to 75% of the global diabetic retinopathy burden.

**Objective:**

The aim of this study was to assess the diagnostic accuracy of diabetic retinopathy screening done by non-ophthalmologists using 2 different digital fundus cameras and to assess the risk factors for the occurrence of diabetic retinopathy.

**Methods:**

This validation study was conducted in 6 peripheral health facilities in Bangladesh from July 2017 to June 2018. A double-blinded diagnostic approach was used to test the accuracy of the diabetic retinopathy screening done by non-ophthalmologists against the gold standard diagnosis by ophthalmology-trained eye consultants. Retinal images were taken by using either a desk-based camera or a hand-held camera following pupil dilatation. Test accuracy was assessed using measures of sensitivity, specificity, and positive and negative predictive values. Overall agreement with the gold standard test was reported using the Cohen kappa statistic (κ) and area under the receiver operating curve (AUROC). Risk factors for diabetic retinopathy occurrence were assessed using binary logistic regression.

**Results:**

In 1455 patients with diabetes, the overall sensitivity to detect any form of diabetic retinopathy by non-ophthalmologists was 86.6% (483/558, 95% CI 83.5%-89.3%) and the specificity was 78.6% (705/897, 95% CI 75.8%-81.2%). The accuracy of the correct classification was excellent with a desk-based camera (AUROC 0.901, 95% CI 0.88-0.92) and fair with a hand-held camera (AUROC 0.710, 95% CI 0.67-0.74). Out of the 3 non-ophthalmologist categories, registered nurses and paramedics had strong agreement with kappa values of 0.70 and 0.85 in the diabetic retinopathy assessment, respectively, whereas the nonclinical trained staff had weak agreement (κ=0.35). The odds of having retinopathy increased with the duration of diabetes measured in 5-year intervals (*P*<.001); the odds of having retinopathy in patients with diabetes for 5-10 years (odds ratio [OR] 1.81, 95% CI 1.37-2.41) and more than 10 years (OR 3.88, 95% CI 2.91-5.15) were greater than that in patients with diabetes for less than 5 years. Obesity was found to have a negative association (*P*=.04) with diabetic retinopathy.

**Conclusions:**

Digital fundus photography is an effective screening tool with acceptable diagnostic accuracy. Our findings suggest that diabetic retinopathy screening can be accurately performed by health care personnel other than eye consultants. People with more than 5 years of diabetes should receive priority in any community-level retinopathy screening program. In a country like Bangladesh where no diabetic retinopathy screening services exist, the use of hand-held cameras can be considered as a cost-effective option for potential system-wide implementation.

## Introduction

Diabetic retinopathy, a progressive eye complication of diabetes mellitus, which affects 9.3% of the people globally, is considered the fifth leading cause of global blindness [1,2]. Results from a recent systematic review estimated that globally, the prevalence of retinopathy among patients with diabetes is 35% [3]. Nearly all patients with type 1 diabetes and more than 60% of the patients with type 2 diabetes develop retinopathy within 20 years of diabetes onset [4]. However, like many other diabetic complications, retinopathy remains asymptomatic until significant damage has occurred [4,5]. Periodic eye screening is essential for diagnosing the disease in early stages and to enable timely initiation of treatment [6]. Current recommendations are that retinal screening occur once a year for all patients with diabetes and that more frequent examinations take place if abnormal findings are identified [7]. However, periodic retinal screening is not available in many low- and middle-income countries although they account for 75% of the global burden of diabetic retinopathy [3,8]. The reported barriers are multifactorial. A recent systematic review reported that poor knowledge and attitudes to asymptomatic eye screening are prevalent both among health care providers and patients [9]. At the health care system level, the lack of equipment, insufficiently skilled professionals, nonfunctioning referral mechanisms, and inadequate data within national management information systems are the main barriers [10]. Moreover, routine diabetic retinopathy screening is not always feasible for eye consultants, given their availability versus the load of patients with diabetes.

As an alternative to eye consultants, different cadres of non-ophthalmologists such as general practitioners, opticians, and diabetologists have been successfully involved in diabetic retinopathy screening in many high-income countries [11]. Particular attention has been focused on developing simple algorithms and technologies suitable for non-ophthalmologists. Among these screening tools, digital fundus photography has been identified as one of the best and lowest cost options [12]. A digital camera allows repeated images to be taken until the best one is captured. Final retinal images can be stored and sent for expert opinion by using a web-based interface [13]. With this technology, high-income countries have shown increased diagnostic accuracy even without pupil dilatation (mydriasis) [12,14]. However, the nonmydriatic approach has shown low accuracy (12%-25%) in the Southeast Asian context [6]. Patients having a dark iris and reporting to a hospital at an advanced age with comorbid eye diseases such as cataracts are the commonly reported explanations for poor vision in the nonmydriatic approach [6].

Like many low- and middle-income countries, Bangladesh demonstrates a substantial diabetic retinopathy disease burden and an array of health system challenges that complicate the routine implementation of diabetic retinopathy screening services. According to the International Diabetic Federation statistics, around 8.4% of the total population in Bangladesh had diabetes in 2017 [15], which puts the country among the top 10 high diabetes burden countries in the world [15]. At the same time, Bangladesh has a critical shortage and “geographic maldistribution” of health workforce with an increased concentration in urban areas even though 70% of the population resides in the rural region [16]. Eye care services are provided predominantly by ophthalmologists, and diabetic retinopathy screening programs are not readily available under the current health system [17]. Given the growing burden of diabetes in Bangladesh, blindness prevention has become a strategic priority [17]. To support this effort, the “Integrated Model of Care for Diabetic Retinopathy within the Health System of Bangladesh” was initiated as a collaborative program between the Fred Hollows Foundation (FHF), a nongovernmental organization and the Government of Bangladesh [18]. This program seeks to establish a care pathway for diabetes and diabetic retinopathy by training non-ophthalmologist health cadres to conduct diabetic retinopathy screening by using digital fundus photography [18].

The aim of this study was to test the diagnostic accuracy of detecting any grade of diabetic retinopathy by non-ophthalmologists using digital fundus cameras against the gold standard diagnosis of ophthalmologists. We also explored the risk factors of diabetic retinopathy in the rural sites under investigation.

## Methods

### Study Setting and Design

This validation study was conducted at the project implementation sites of FHF in 4 districts under 2 administrative divisions of Bangladesh. Six health facilities were randomly chosen from these 4 districts for our study: one medical college hospital, one district hospital, and 4 health centers of the Diabetic Association of Bangladesh (DAB). The medical college hospital and the district hospital are government-funded general hospitals that provide eye care services for patients with or without diabetes. DAB centers are autonomous health care organizations focused on the treatment of patients with diabetes only. Retinopathy screening for asymptomatic cases is not a regular clinical care option in any of these health facilities. Eye consultants were available in all government hospitals and in 1 out of the 4 DAB centers. As part of project activities, the FHF established a memorandum of understanding with the concerned health facilities to establish a diabetic retinopathy screening corner within the eye department to provide equipment supplies and to organize relevant local and national-level trainings on diabetic retinopathy diagnosis and treatment for hospital staff. A parallel referral mechanism linking DAB centers and the closest public health facility was also established.

### Diabetic Retinopathy Screening Process

Patients with diabetes attending the outdoor eye clinic at the selected study health facilities between July 2017 and June 2018 were included as the study participants. Two different types of digital fundus photography instruments were used for diabetic retinopathy screening: a desk-based high-resolution fundus camera and a hand-held low-cost fundus camera. Initial screening was done by non-ophthalmologists such as nurses, paramedics, and nonclinical trained staff. For the gold standard diagnosis, 2 eye consultants, one from each study division, were assigned to evaluate the screening done by the non-ophthalmologists for the respective administrative divisions. The desk-based camera is used in medical colleges and district hospitals, and registered nurses and paramedics are the primary diabetic retinopathy screening providers. Hand-held cameras are used in DAB centers. Other than 1 DAB center, none could deploy their own nurses/paramedics due to the high turnover rate. Thus, new project staff were recruited from FHF, one in each of the 3 DAB centers. These were nonclinical personnel with graduate degrees in any discipline. All the nonclinical staff, including nurse and paramedics, received hands-on training for 3 days from the eye consultant in the respective study division. Nurses working in the medical college obtained an opportunity to attend a month-long training at the national level. Once the 2 eye consultants certified that images taken by the non-ophthalmologists were satisfactory and their ability to detect diabetic retinopathy from the retinal images was accurate, the formal data collection process started.

Initially, the non-ophthalmologist staff took the retinal images and performed diabetic retinopathy grading independently. A short-acting pupil dilator was used prior to taking the retinal image and then, a single-field macula-focused image was taken. Subsequently, the same study participant with a referral slip (indicating patient ID and date of diabetic retinopathy screening) was referred to the eye department of the respective district or medical college hospital to be examined by the eye consultant. Retinal images were also sent to the eye consultant through a web-based interface or a portable computer disk. The eye consultants checked the gradeability of the retinal images provided through the web-based interface and performed diabetic retinopathy grading independently. The entire screening process was double-blinded, that is, no one had access to the findings of the others. The project-appointed staff monitored the data collection and retrieved data from the hospital records with a diagnostic accuracy checklist.

### Sampling Strategy

The inclusion criteria were patients with type 1 diabetes older than 18 years or with type 2 diabetes having no previous diagnosis of diabetic retinopathy and images taken from both eyes. Exclusion criteria were patients with significant physical or mental disabilities that could hamper photography and having mature cataract and corneal opacity in any eye. Considering the current facility-based prevalence of diabetic retinopathy as 27% [19], a sample size calculation was performed for sensitivity and specificity. The final sample size was the maximum of these two [20]. For the anticipated sensitivity and specificity, we considered the Canadian and British Ophthalmology Society guidelines. Both guidelines recommend at least 80% sensitivity and 90%-95% specificity for any alternative approach of diabetic retinopathy grading [21,22]. Taking all these into account, our required sample size was 1138, and we distributed them proportionately across the 6 study health facilities, considering the patient turnover rate.

### Outcome Measures

The outcome variable was the presence of any form of diabetic retinopathy in either eye of a patient, which was confirmed by the eye consultant. The Airlie house classification was used for diabetic retinopathy staging, which is a validated tool for the diabetic retinopathy screening program [22]. This classification divides the diagnosis of diabetic retinopathy into 5 stages: no diabetic retinopathy, mild nonproliferative diabetic retinopathy, moderate nonproliferative diabetic retinopathy, severe nonproliferative diabetic retinopathy, and proliferative diabetic retinopathy. A diabetic retinopathy positive case referred to a patient who had any kind of diabetic retinopathy (mild nonproliferative diabetic retinopathy/moderate nonproliferative diabetic retinopathy/severe nonproliferative diabetic retinopathy/proliferative diabetic retinopathy) in any of the two eyes.

### Covariates

We considered the patient’s sociodemographic and clinical characteristics, both of which have been identified as risk factors of diabetic retinopathy in the literature [3]. Among the sociodemographic features, patient age, gender, education, and occupation were considered. Clinical covariates were BMI, duration of diabetes, recent blood sugar test result, and presence of hypertension. BMI was calculated from height and weight measurements performed on the day of the clinic visit by using the following formula: weight in kilograms/height in meters squared. The World Health Organization criteria of BMI classification for Asian people was used to categorize our sample into 4 groups [23]. For diabetes test results, we considered any form of blood sugar test done within 3 months with written documentation provided by the patient during diabetic retinopathy screening. We categorized diabetes test results into normal limit and uncontrolled blood sugar by using reference values provided by the available blood glucose tests [24].

### Statistical Analysis

We calculated the test accuracy of non-ophthalmologists against the gold standard value and reported measures of sensitivity (true positive diabetic retinopathy/[true positive + false negative]) and specificity (true negative diabetic retinopathy/[true negative + false positive cases]) at 95% CI. The positive predictive values and negative predictive values were also calculated from the true diabetic retinopathy positive and diabetic retinopathy negative results out of the total positive and negative test results, respectively [25]. All diagnostic accuracy results were compared by instrument types (desk-based camera vs hand-held camera). Additionally, differences in diagnostic accuracy were measured across the different diabetic retinopathy grades. Overall agreement and disagreement were tested using the Cohen kappa statistic and area under the receiver operating curve (AUROC). The AUROC is an index of accuracy [26] presented as a plot of true positive rates against false positive rates for different possible cut-off points of a diagnostic test [26]. Descriptive analysis reports the distribution of the study sample by covariates. The statistical association of diabetic retinopathy positive status with all covariates were tested initially through a bivariate analysis using the chi-square test. Covariates that were significant at a *P* value <.05 in bivariate analysis were included in the multivariate analysis. Binary logistic regression findings were presented as odds ratio (OR) at 95% CI. All the analyses were performed using the STATA software (Release 16, College Station, StataCorp LLC) [27].

### Ethics Approval

We obtained ethical clearance from the Institutional Review Board of icddr,b, Dhaka, Bangladesh under protocol number 17003. Verbal informed consent was obtained from respective hospital authorities and study participants to access medical records. Data collected from the study participants were reported anonymously to maintain privacy and confidentiality. Study participants with poor-quality retinal images or confusing findings were referred to an eye consultant for further evaluation, and transport costs were remunerated. Any patient requiring laser treatment obtained this service from a tertiary-level public hospital free of cost.

## Results

### Characteristics of the Participants in This Study

In total, 1511 patients with diabetes were screened, which was slightly higher than our required sample size, and we included all of them. However, 3.7% (56/1511) of the images (including both eyes) were of poor quality and discarded from analysis. Thus, our final analytic sample consisted of 1455 patients. The mean (SD) age of the study participants was 53.23 (11.84) years, and a slightly larger proportion of the total study participants was females. More than half of the study participants had diabetes for more than 5 years. Blood sugar test results were missing for 7.0% (102/1455) of the patients. Blood sugar levels were reported 2 hours after breakfast in 52.5% (764/1455) of the patients and <1% (2/1455) of the patients by the hemoglobin A_1C_ test. The mean BMI of the patients was 25.38 (4.21). Nearly half of the patients with diabetes were screened by a nurse, and half were assessed using a desk-based camera (Table 1).

**Table 1:** Characteristics of the study participants who attended diabetic retinopathy screening from July 2017 to June 2018 in 6 selected peripheral hospitals in Bangladesh (N=1455).

Variable of interest		Values, n (%)
**Patient age (years)**		
	<40 years	247 (16.98)
	41-50 years	399 (27.42)
	51-60 years	471 (32.37)
	>60 years	338 (23.23)
**Gender**		
	Female	814 (55.95)
	Male	641 (44.05)
**Education**		
	No schooling	256 (17.59)
	Primary school completed	309 (21.24)
	Higher secondary and above	890 (61.17)
**Occupation**		
	Unemployed	1001 (68.80)
	Service	266 (18.28)
	Business	188 (12.92)
**Body mass index**		
	Normal and underweight	429 (29.71)
	Overweight	632 (43.77)
	Obese	383 (26.52)
**Duration of diabetes**		
	<5 years	513 (35.26)
	5-10 years	459 (31.55)
	>10 years	483 (33.19)
**Blood sugar level**		
	Controlled	613 (45.31)
	Not controlled	740 (54.69)
**Hypertensive patient**		
	No	659 (45.29)
	Yes	796 (54.71)
**Type of non-ophthalmologist**		
	Nurse	766 (52.65)
	Paramedics	276 (18.97)
	Nonclinical trained staff	413 (28.38)
**Instrument used**		
	Hand-held camera	576 (39.59)
	Desk-based camera	879 (60.41)
**Place of training for non-ophthalmologists**		
	Local	824 (56.63)
	National	631 (43.37)

### Diabetic Retinopathy Accuracy Test Results

The prevalence of diabetic retinopathy was 38.39% (558/1455). As shown in Table 2, the diagnostic accuracy of non-ophthalmologists was 86.6% (483/558, 95% CI 83.5%-89.3%) for diabetic retinopathy positive case detection (sensitivity) and 78.6% (705/897, 95% CI 75.8%-81.2%) for diabetic retinopathy negative case detection (specificity). Further, non-ophthalmologists could correctly identify 71.6% (483/675) of the total diabetic retinopathy positive cases and correctly exclude 90.4% (705/780) of the total diabetic retinopathy negative cases. As the kappa value suggested, moderate agreement was observed with the gold standard (κ=0.6).

**Table 2:** Diagnostic accuracy of diabetes retinopathy by instrument type in 6 selected peripheral hospitals in Bangladesh from July 2017 to June 2018.

Indicators	Overall proportion (%) 95% CI	Hand-held camera proportion (%) 95% CI	Desk-based camera proportion (%) 95% CI
Sensitivity	86.56 (83.45-89.30)	85.60 (80.30-89.89)	87.19 (83.19-90.60)
Specificity	78.60 (75.78-81.2)	56.56 (51.19-61.70)	93.01 (90.50-95.01)
Positive predictive value	71.56 (68.78-74.10)	55.19 (51.89-58.40)	88.50 (84.89-91.30)
Negative predictive value	90.38 (88.39-92.10)	86.19 (81.70-89.67)	92.19 (89.89-93.89)
Accuracy	81.56 (79.56-83.60)	67.70 (63.70-71.65)	90.78 (88.70-92.56)
Kappa	0.63 (0.58-0.78)	0.38 (0.31-0.46)	0.80 (0.74-0.87)

**Figure 1:**
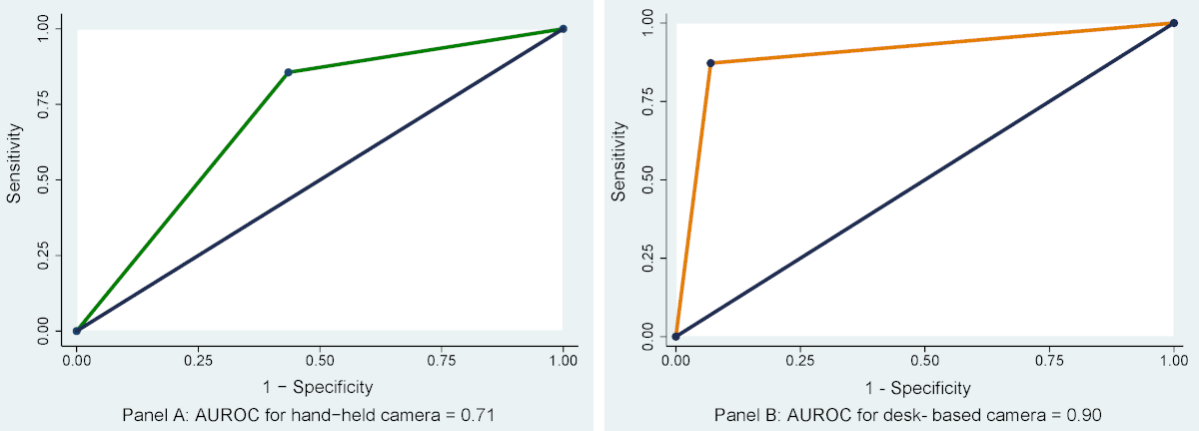
Diagnostic accuracy of digital fundus photography by area under receiver operating curve. Panel A: hand-held camera and Panel B: desk-based camera.

Accuracy was further reported by AUROC findings. As shown in Figure 1, the ability of non-ophthalmologists to correctly classify diabetic retinopathy by using a hand-held camera was “Fair” (AUROC 0.710, 95% CI 0.67-0.74) (Figure 1, Panel A) and “Excellent” (AUROC 0.901, 95% CI 0.88-0.92) by using a desk-based camera (Figure 1, Panel B). We also assessed the agreement of different cadres of non-ophthalmologists against the gold standard value. Strong agreement was noted between the diabetic retinopathy classification of registered nurses and paramedics and that of the gold standard diagnosis by eye consultants, with kappa values of 0.70 and 0.85, respectively, whereas nonclinical trained staff had weak agreement (κ=0.35). As shown in Table 3, non-ophthalmologists were particularly good at detecting the presence or absence of diabetic retinopathy. However, their accuracy differed by the diabetic retinopathy grading status particularly across the different grades of nonproliferative diabetic retinopathy. Accuracy also varied slightly depending on the type of instrument used. A desk-based camera was more likely to identify diabetic retinopathy correctly when diabetic retinopathy was present than when it was absent. In contrast, the probability of a correct diabetic retinopathy diagnosis was lower among those with a negative diabetic retinopathy finding versus those without. Although the hand-held camera was less successful in identifying diabetic retinopathy correctly in the presence of diabetic retinopathy, the probability that a person showed a negative finding with a hand-held camera for diabetic retinopathy was lower than that with a desk-based camera.

**Table 3:** Diagnostic accuracy of diabetic retinopathy screening by instrument types across different diabetic retinopathy stages in 6 selected peripheral hospitals in Bangladesh from July 2017 to June 2018.

Stage of diabetic retinopathy	Sensitivity/specificity (%) of hand-held camera	Sensitivity/specificity (%) of desk-based camera
No diabetic retinopathy	85.59 (56.50)	87.20 (93.00)
Mild NPDR^a^	69.35 (62.17)	59.26 (92.09)
Moderate NPDR	50.75 (92.53)	49.02 (96.91)
Severe NPDR	52.00 (96.37)	72.00 (93.03)
PDR^b^	33.33 (97.19)	49.02 (99.52)

^a^NPDR: nonproliferative diabetic retinopathy.

^b^PDR: proliferative diabetic retinopathy.

### Determinants of Diabetic Retinopathy

Table 4 shows the determinants of diabetic retinopathy identified through bivariate and logistic regression analyses. Patient’s age, education, BMI, diabetes duration, and controlled blood sugar levels were significant factors at 5% significance level while predicting diabetic retinopathy occurrence. After controlling for all significant covariates from bivariate results, the probability of diabetic retinopathy was found to increase with increasing educational level, BMI, duration of diabetes for more than 5 years, and uncontrolled blood sugar levels. A graded response was observed between longer duration of diabetes and probability of diabetic retinopathy occurrence. However, BMI showed a negative association with diabetic retinopathy; the odds of having diabetic retinopathy decreased significantly with high BMI (OR 0.59, 95% CI 0.43-0.81) compared to the reference group consisting of normal weight and underweight patients. 

**Table 4:** Factors associated with diabetic retinopathy occurrence in Bangladesh from July 2017 to June 2018.

Variable of interest		No diabetic retinopathy^a^ (n=897), n (%)	Diabetic retinopathy present (n=558), n (%)	*P* value	Adjusted effect odds ratio (95% CI)	*P* value
**Patient age (years)**				.003^a^		
	<40 years	174 (70.5)	73 (29.6)		Ref^b^	
	41-50 years	236 (59.1)	163 (40.9)		1.49 (1.03-2.16)	.03^a^
	51-60 years	270 (57.3)	201 (42.7)		1.32 (0.91-1.90)	.14
	>60 years	217 (64.2)	121 (35.8)		0.99 (0.67-1.48)	.97
**Gender**				.09		
	Male	380 (59.3)	261 (40.7)		N/A^c^	N/A
	Female	517 (63.5)	297 (36.5)		N/A	N/A
**Education**				.001^a^		
	No schooling	185 (72.3)	71 (27.7)		Ref	
	Primary completed	186 (60.2)	123 (39.8)		1.48 (1.00-2.19)	.05^a^
	≥Higher secondary	526 (59.1)	364 (40.9)		1.45 (1.03-2.03)	.03^a^
**Occupation**				.43		
	Unemployed	628 (62.7)	373 (37.3)		N/A	N/A
	Service	159 (59.8)	107 (40.2)		N/A	N/A
	Business	110 (58.5)	78 (41.5)		N/A	N/A
**Body mass index**				.04^a^		
	Normal and underweight	254 (59.2)	175 (40.8)		Ref	
	Overweight	378 (59.8)	254 (40.2)		0.92 (0.70-1.22)	.58
	Obese	256 (66.8)	127 (33.2)		0.59 (0.43-0.81)	.001^a^
**Duration of diabetes**				<.001^a^		
	<5 years	395 (77.0)	118 (23.0)		Ref	
	5-10 years	292 (63.6)	167 (36.4)		1.98 (1.47-2.66)	<.001^a^
	>10 years	210 (43.5)	273 (56.5)		4.36 (3.23-5.89)	<.001^a^
**Diabetes controlled**				<.001^a^		
	Yes	410 (66.9)	203 (33.1)		Ref	
	No	418 (56.5)	322 (43.5)		1.45 (1.15-1.84)	.002^a^
**Hypertensive patient**				.27		
	No	501 (62.9)	295 (37.1)		N/A	N/A
	Yes	396 (60.1)	263 (39.9)		N/A	N/A

^a^Results are significant at *P*<.05.

^b^Ref: reference category, that is, <40 years, no schooling, normal and underweight category of BMI, diabetes duration <5 years and diabetes controlled for the corresponding covariates. Diabetic retinopathy present was the reference value for the outcome variable.

^c^N/A: not applicable. Implies not included in the multivariate model.

## Discussion

Conducted in 6 peripheral health facilities in Bangladesh, this validation study showed that non-ophthalmologists can detect the presence of diabetic retinopathy with reasonable diagnostic accuracy. The sensitivity and specificity of using a single-field macular photograph after pupillary dilatation against the referral gold standard diagnosis by an eye consultant were 86.6% (483/558) and 78.6% (705/897), respectively. The degree of diagnostic agreement between a non-ophthalmologist and an ophthalmologist was excellent with a desk-based camera and satisfactory with a hand-held camera.

To our knowledge, this is the first ever study in Bangladesh to engage health care personnel other than eye consultants in routine retinal examination. The reported true positive diabetic retinopathy case detection rate was within the international standard of more than 80% [21,22]. However, our overall specificity (79%) was lower than the international recommendation of 90%-95% [21,22]. This implies that 19.9% (173/869) of the total diabetic retinopathy negative cases were referred to an eye consultant when they did not have diabetic retinopathy [26]. In generic terms, lower specificity implies greater health system burden with a greater number of false positive cases referred to the next level [26]. Nevertheless, in a country like Bangladesh where no formal diabetic retinopathy screening services are available, a large proportion of people with diabetes remain undiagnosed until opportunistic diagnosis occurs at a very advanced stage [28]. Considering the increasing burden of diabetic retinopathy, this will result in increasing eye care–related costs and a greater risk of blindness [29]. By training and engaging non-ophthalmologist health staff in diabetic retinopathy screening, limited resources can be maximized and coverage increased [25]. However, evidence to support the incorporation of non-ophthalmologists into the diabetic retinopathy screening pathway remains scarce in low- and middle-income settings [30]. Some studies in India, Sri Lanka, and Pakistan report satisfactory test accuracy for non-ophthalmologist screening [6,9,31,32]. Using digital fundus photographs and a nonmydriatic approach, “physician graders” in Sri Lanka showed a sensitivity of 88.7% and specificity of 94.9% [10]. In Pakistan, optometrists showed 72% sensitivity and 86.3% specificity [32]. A decision must also be made about which instrument and imaging technique to use and whether to dilate or not dilate [33]. We validated the diagnostic agreement of hand-held versus desk-based cameras by calculating the AUROC and kappa values. The AUROC was “close to 1” for both instruments [26], suggesting a fair-to-excellent amount of agreement. However, in low- and middle-income country settings, instrument costs should also be considered. Here, the hand-held camera performs better—being comparatively less expensive than a desk-based camera and easier to carry and employ in the context of community-level screening [34].

The variation in test accuracy by instrument type further emphasizes the need to explore other factors influencing diagnostic test accuracy. Here, the gradeability of images plays an important part in successful diabetic retinopathy screening. In this study, a lower rate of ungradable images was reported (56/1511, 4.1%) than that reported in similar health facility–based studies in the South Asian context. A technical failure rate of 7.5% was noted in another study in Bangladesh [35], which rose to 12% in Sri Lanka [10]. The higher level of gradeability observed in our study is probably a function of the exclusion criteria or the image-taking technique that was employed. Considering the higher prevalence of cataracts in low-income country settings [29,36], we made the presence of cataract an exclusion criterion in our sample selection and chose to deploy a mydriatic approach for imaging. Although the gold standard for diabetic retinopathy screening is a 7-field photograph using a nonmydriatic approach [37], the applicability of this technique in low-income country settings is highly controversial [38]. When using a nonmydriatic approach, the body’s autonomic nervous system becomes hyperactive with reflex pupillary constriction in the second eye after taking an image of the first eye [39,40]. Further, a Brazilian study showed that longer screening time and more referrals to ophthalmologists occurred in the absence of pupil dilation due to a larger number of ungradable images [41]. Further supporting our approach is the evidence of increased provider compliance when using a single-field photograph [42].

To identify other factors affecting diabetic retinopathy diagnosis, we compared test accuracy across different cadres of non-ophthalmologist personnel. We observed that nurses and paramedics showed higher accuracy in detecting any form of diabetic retinopathy than the nonclinical trained staff [43]. The relatively poorer performance of the nonclinical trained staff may be due to their lack of comfort with the hand-held camera. We expect that with more onsite supportive training, performance can be increased. Supporting the call for more hands-on training, a study suggested that the diagnostic accuracy of the fundus camera is highly dependent on the user’s technique in taking a correct image and their ability to do proper grading—both of which can be improved with more hands-on support [44]. Variations in the test accuracy by type of provider were similarly observed in a systematic review of 22 observational studies [45]. Noting that the sensitivity of detecting any form of retinopathy using a mydriatic approach ranged between 87% and 100% for general practitioners, >91% for optometrists, and 89% and 93% for ophthalmologists or their assistants, the authors of a study concluded that using appropriate technology and ensuring quality are more important than the type of provider in diabetic retinopathy screening programs [45].

We also explored factors predicting retinopathy occurrence among patients with diabetes. Increased duration of diabetes and obesity were identified as significant predictors of diabetic retinopathy. The odds of having diabetic retinopathy was found to increase 2 times among patients with diabetes for 5-10 years and 4 times among those with diabetes for more than 10 years compared to the odds of having diabetic retinopathy in those with diabetes for less than 5 years. While the increased duration of diabetes for the development of diabetic retinopathy is well-established [3,8,46,47], the observed negative association between obesity and diabetic retinopathy in this study was inconsistent with that reported in the literature. Only 1 study conducted in an urban slum in India reported a similar result; those in the overweight category had lower odds of diabetic retinopathy (OR 0.6, 95% CI 0.4-0.9) than people with normal BMI [31]. In general, however, obesity in the Asian context has been identified as a risk factor for developing diabetic retinopathy [1].

Although results from this pilot study are supportive of mainstreaming non-ophthalmologist health staff in diabetic retinopathy screening services, some limitations in the diagnostic processes need to be considered before generalizing this recommendation to other contexts. One important limitation was our decision to exclude patients with cataract and to employ a mydriatic and single-field macular photographic approach due to the pilot nature of this research. In this regard, further clinical trials may be useful for determining the test accuracy by type of health professional, type of instrument used, use of pupil dilation, and number of fields chosen for screening. Finally, recommendations emerging from a study conducted in hospitals with specialized diabetes or eye departments may be less pertinent in nonspecialist hospitals where the prevalence of diabetic retinopathy is likely lower.

Considering the rising burden of diabetes in Bangladesh, routine retinal screening of patients with diabetes is not feasible by eye consultants alone. Our study results suggest that non-ophthalmologist staff such as nurses and paramedics could function as frontline health workers in diabetic retinopathy screening programs. As the first step, the engagement of non-ophthalmologist cadres in diabetic retinopathy screening should be limited to categorization based on the presence or absence of diabetic retinopathy only. Their involvement in the more technical area of diabetic retinopathy grading requires further specific training and health system level support. Regarding instrument choice, although the desk-based camera shows better accuracy in detecting diabetic retinopathy, the choice of instrument type should be a function of the capacity of the health facility and the health care provider performing the diagnosis.

## Abbreviations

AUROC: area under the receiver operating curve
DAB: Diabetic Association of Bangladesh
FHF: Fred Hollows Foundation
OR: odds ratio
